# Preoperative sarcopenia and postoperative accelerated muscle loss negatively impact survival after resection of locally advanced gastric cancer

**DOI:** 10.1186/s12885-025-13674-3

**Published:** 2025-02-14

**Authors:** Xiaolong Li, Ping’an Ding, Jiaxiang Wu, Haotian Wu, Peigang Yang, Honghai Guo, Yuan Tian, Lingjiao Meng, Qun Zhao

**Affiliations:** 1 The Third Department of Surgery, The Fourth Hospital of Hebei Medical University, Shijiazhuang, Hebei 050011 China; 2 Hebei Key Laboratory of Precision Diagnosis and Comprehensive Treatment of Gastric Cancer, Shijiazhuang, 050011 China; 3 Big Data Analysis and Mining Application for Accurate Diagnosis and Treatment of Gastric Cancer, Hebei Provincial Engineering Research Center, Shijiazhuang, 050011 China; 4 Department of General Surgery, Baoding First Central Hospital, Baoding, Hebei 071000 China; 5 Research Center of the Fourth Hospital of Hebei Medical University, Shijiazhuang, 050011 China

**Keywords:** Stomach neoplasm, Sarcopenia, Skeletal muscle loss, Prognosis, Radical surgery

## Abstract

**Background:**

Gastric cancer remains a major health concern worldwide, with locally advanced gastric cancer (LAGC) posing significant challenges due to frequent lymph node metastasis and poor prognosis. While curative gastrectomy with D2 lymph node dissection is the standard treatment, sarcopenia and perioperative skeletal muscle loss (SML) have emerged as critical prognostic factors.

**Methods:**

We retrospectively analyzed 198 LAGC patients who underwent curative gastrectomy. Preoperative and postoperative sarcopenia were assessed via computed tomography (CT)-derived skeletal muscle index (SMI) at the L3 level. SML was defined based on sex-specific thresholds of SMI changes (%/30 days). Prognostic significance for overall survival (OS) and disease-free survival (DFS) was evaluated using Kaplan-Meier and Cox regression analyses.

**Results:**

The prevalence of sarcopenia increased from 23.7% preoperatively to 33.3% postoperatively. Patients with significant muscle loss (SML) showed worse OS and DFS compared to non-SML patients (*P* < 0.05). SML was also associated with a higher incidence of Clavien-Dindo grade ≥ 3a complications (*P* < 0.05). Multivariate analysis identified preoperative sarcopenia (HR = 2.332, *P* = 0.001), postoperative sarcopenia (HR = 3.189, *P* = 0.011), and SML (HR = 11.231, *P* = 0.002) as independent risk factors for OS. Adjuvant chemotherapy significantly improved both OS (HR = 0.532, *P* = 0.015) and DFS (HR = 0.587, *P* = 0.041).

**Conclusions:**

Both preoperative sarcopenia and perioperative SML may negatively impact postoperative prognosis in LAGC patients, suggesting that perioperative evaluation of skeletal muscle mass may help identify high-risk surgical candidates for targeted interventions.

**Supplementary Information:**

The online version contains supplementary material available at 10.1186/s12885-025-13674-3.

## Introduction

Gastric cancer (GC) is one of the most common malignancies worldwide [1–2]. Locally advanced gastric cancer (LAGC), characterized by tumor invasion beyond the submucosal layer and frequent lymph node metastasis (LNM), presents significant challenges in treatment [3–4]. While curative gastrectomy with D2 lymph node dissection (D2), which involves the removal of both perigastric and additional lymph nodes along the celiac axis, common hepatic artery, and splenic artery, remains the standard treatment, high rates of postoperative complications and poor survival outcomes highlight the need for improved prognostic strategies [5–6]. Sarcopenia, defined as the progressive loss of skeletal muscle mass and function, has emerged as an important prognostic factor in cancer patients [7–8]. Preoperative sarcopenia has been linked to increased postoperative complications, prolonged hospitalization, and reduced overall survival (OS) and disease-free survival (DFS) [9–10]. Despite its recognized significance, the specific impact of perioperative skeletal muscle loss (SML) on the clinical outcomes of LAGC patients undergoing curative surgery remains underexplored. While previous studies have evaluated sarcopenia’s role in various malignancies, including colorectal [11–12] and pancreatic cancers [13–14], few have focused on its perioperative dynamics in LAGC patients. This gap in knowledge is particularly relevant, as LAGC patients often present with malnutrition and systemic inflammation, which may exacerbate muscle wasting and worsen surgical outcomes.

Recent advancements in imaging technology, particularly the use of computed tomography (CT) for body composition analysis, have provided a robust and non-invasive method to assess skeletal muscle mass [15–17]. However, the diagnostic criteria for sarcopenia remain inconsistent across studies, and limited research has addressed sex-specific differences in muscle loss thresholds. Furthermore, most studies have concentrated on baseline preoperative sarcopenia, with minimal attention to the temporal changes in skeletal muscle mass during the perioperative period [18–20]. This is a critical oversight, as perioperative SML may serve as a dynamic marker of patient frailty and a predictor of long-term outcomes.

To our knowledge, this study represents one of the first comprehensive evaluations of the perioperative changes in skeletal muscle mass in LAGC patients undergoing curative gastrectomy. By quantifying SML through serial CT scans and correlating these changes with postoperative complications, OS, and DFS, this study provides a novel perspective on the prognostic significance of muscle loss in LAGC. Our findings highlight the importance of identifying patients at high risk of SML and implementing targeted interventions, such as personalized nutritional and rehabilitation programs, to mitigate its impact. In addition, this study introduces sex-specific thresholds for SML, derived from receiver operating characteristic (ROC) curve analyses, offering a more tailored approach to sarcopenia assessment. By addressing the limitations of previous research and focusing on the dynamic nature of skeletal muscle changes, our work aims to bridge the gap between sarcopenia diagnosis and clinical application, ultimately improving the management and outcomes of LAGC patients.

## Materials and methods

### Patients

We retrospectively reviewed two prospective clinical studies of LAGC (NCT01516944, NCT02555358) to identify curative gastrectomy-treated adult patients between May 2011 and June 2018 at the Fourth Hospital of Hebei Medical University. Patients were included if: (I) ages ≥ 18 years; (II) diagnostically confirmed GC; (III) no pre-surgical anticancer therapy, and (IV) the Eastern Cooperative Oncology Group (ECOG) scores were ≤ 2 points. The exclusion criteria included: (I) residual cancer cells around R1/R2 resection margins; (II) incomplete/missing clinical data; (III) existing other types of tumors and/or hematological diseases; (IV) abnormal blood routine due to pre-op infections, and (V) obstructed skeletal measurement at L3 due to metal implant in the lumbar area. This cohort study was conducted under the approval of the Medical Ethics Committee of the Fourth Hospital of Hebei Medical University (Approval No. 2024KY198) following the recommendations of the Declaration of Helsinki. As this was a retrospective study that did not involve the disclosure of patient privacy and posed minimal risk, the requirement for informed consent was waived by the ethics committee.

### Data collection and follow-ups

Data on preoperative clinical parameters [gender, age, body mass index (BMI), albumin (Alb) level, and pre-op comorbidity], prognostic nutritional index (PNI), pre-/post-op skeletal muscle index (SMI), operative features (method and duration of surgical resection, and volume of intraoperative blood loss), post-op complications, tumor-node-metastasis (TNM) staging, and diagnosis of pathology. Post-op adverse events were graded by the Clavien-Dindo (CD) classification system^[13]^, and a grade of ≥ 3a indicated severe complications. Age was categorized using a cut-off value of 50 years based on clinical relevance, previous literature, and univariate analysis results demonstrating significant prognostic differences in survival outcomes between the age groups [21–22]. BMI was classified according to WHO criteria and Asian-specific BMI guidelines. Patients with BMI < 18.5 kg/m^2^ were categorized as underweight, while those with BMI ≥ 18.5 kg/m^2^ were considered to have normal or higher BMI.

OS and DFS were respectively set as primary and secondary endpoints. Time to the endpoint (Days) was determined by subtracting the date of surgery from that of the event. Causes of death were classified as gastric cancer-related, other cancer-related, and non-cancer-related. Recurrence patterns were categorized as locoregional recurrence, distant metastasis (liver, lung, or bone), and peritoneal recurrence. Data were collected from follow-up records and imaging reports.

### Adjuvant chemotherapy regimen

Adjuvant chemotherapy was indicated for patients with LAGC following curative gastrectomy, specifically those with stage II or III disease based on the AJCC 8th edition TNM staging system. Patients with stage I disease or those who were deemed unfit for chemotherapy due to poor general condition (e.g., ECOG performance status > 2 or severe comorbidities) were excluded from adjuvant therapy. Adjuvant chemotherapy was initiated 4 to 6 weeks after gastrectomy in patients with stage II or III disease who demonstrated adequate postoperative recovery. The regimens used included XELOX (oxaliplatin plus capecitabine) and SOX (oxaliplatin plus S-1). SMI was measured at one month postoperatively, prior to the initiation of adjuvant chemotherapy for most patients.

### Computed tomography (CT) evaluation

The pre-op baseline CT was done one week before surgery, and the post-op follow-up examination was performed after one month of curative gastrectomy and before the first adjuvant therapy session.

All the flat-scan (5-mm) images were deposited to the PACS (SIEMENS SOMATOM). Sarcopenia was evaluated using axial CT, and the cross-sectional muscle areas (cm^2^) indicated changes in the muscle quantity at the level of the inferior endplate of L3. At L3, the total cross-sectional area of the psoas, bilateral paraspinal, and abdominal wall muscles was assessed and segmented using the multi-modal syngo.via software (SIMENS) [23]. The SMI was calculated by setting the lean muscle threshold range from − 29 to 150 HU. The image analysis investigator was kept blind to the patient’s outcome results to ensure no bias in measurements and calculations. For individual subjects, SMI was normalized by dividing the total muscle cross-sectional area (cm^2^) by the square of the patient’s height (m^2^) [24]. The CT images of pre-op baseline and post-op follow-up were used to quantify the change in the skeletal muscle mass, and the corresponding change in SMI (ΔSMI) was expressed as the percent (%) change per 30 days using the formula (SMI^post−op^– SMI^pre−op^)/ SMI^pre−op^ × 100.

For female subjects, sarcopenia was confirmed when SMI < 34.9 cm^2^/m^2^, while for male patients, it was < 40.8 cm^2^/m^2^, per the Asian Working Group for Sarcopenia (AWGS) guidelines [25].

### Statistical analyses

SPSS v21.0 software (SPSS Inc., USA) was employed for all statistical analyses. Graphs were prepared on the GraphPad Prism v8.01 (GraphPad Inc., USA). Normally distributed continuous variables were calculated as the mean ± standard deviation (SD), while non-normally distributed variables as the median and inter-quartile range (IQR). Categorical variables were presented as the number (n) and percentage (%). The independent *t*-test and Mann-Whitney U (MN-U) test were used for comparing continuous variables, and for categorical variables, the chi-squared (χ^2^), and Fisher’s exact tests were utilized. Patients were classified as good or worse OS based on the threshold value of ΔSMI(%)/30 days, which was obtained from the receiver operating characteristic (ROC) curve analysis for the highest Youden’s index. OS and DFS were evaluated by Kaplan-Meier survival analysis and the log-rank test. Variables with *P* < 0.05 in univariate analysis were included in the multivariate Cox proportional hazards model. The hazard ratios (HR) and 95% confidence intervals (CI) were calculated to identify independent risk factors for OS and DFS. A P-value of < 0.05 referred to the statistical significance.

## Results

### Study population

**Fig. 1:**
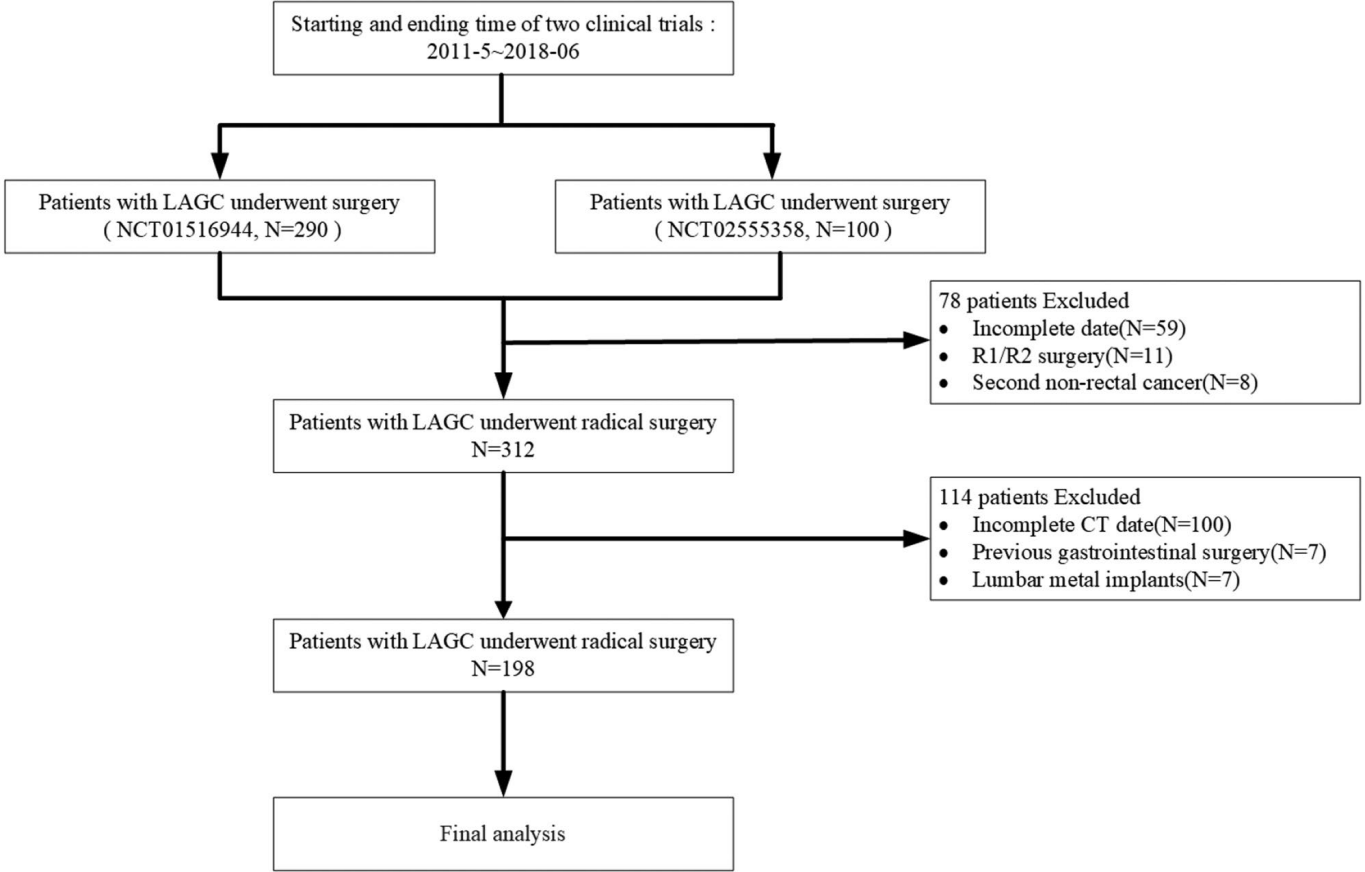
Flowchart presentation of patient selection, enrollment, and exclusion criteria

After final selection, 198 LAGC cases (132 men; 66.7%) were enrolled in this study. The mean age of the study cohort was 58.9 years (Fig. 1). Patients with pre-op sarcopenia had lower BMI (pre-: 23.2 ± 2.2 vs. 25.1 ± 2.5 kg/m^2^; *P* < 0.001; post-: 21.4 ± 2.5 vs. 23.6 ± 2.6 kg/m^2^; *P* < 0.001), SMI (pre-: 38.9 ± 5.2 vs. 47.8 ± 7.6 cm^2^/m^2^; *P* < 0.001; post-: 36.6 ± 5.3 vs. 43.4 ± 7.2 cm^2^/m^2^; *P* < 0.001), and PNI (pre-: 53.1 ± 6.7 vs. 55.1 ± 5.4; *P* < 0.001; post-: 52.5 ± 5.2 vs. 53.5 ± 5.1; *P* < 0.001) than those without. When stratified by age, the pre-op sarcopenia candidates had higher mean age than that of nonsarcopenic subjects (64.7 ± 12.3 vs. 57.0 ± 11.5 years; *P* < 0.001), and their clinical features were independent of sex difference. The baseline characteristics of LAGC subjects are described in Table 1.

**Table 1:** Demographics and postoperative outcomes based on the respective preoperative SMI

Clinical features	Non-sarcopenia (*N* = 151)	Sarcopenia (*N* = 47)	*P* value
**Age (years)**	57.0 ± 11.5	64.7 ± 12.3	< 0.001
**Sex (n**,** %) Male**	97 (64.2)	35 (74.5)	0.194
**BMI (kg/m** ^**2**^ **)**			
Preoperative	25.1 ± 2.5	23.2 ± 2.2	< 0.001
Postoperative	23.6 ± 2.6	21.4 ± 2.5	< 0.001
**PNI**			
Preoperative	55.1 ± 5.4	53.1 ± 6.7	< 0.001
Postoperative	53.5 ± 5.1	52.5 ± 5.2	< 0.001
**ASA classification (n**,** %)**			0.009
I	87 (57.6)	15 (31.9)	
II	60 (39.7)	30 (63.8)	
III or higher	4 (2.6)	2 (4.3)	
**Surgical approach (n**,** %)**			0.095
Open	115 (76.2)	30 (63.8)	
Laparoscopy	36 (23.8)	17 (36.2)	
**Operation time (min)**	188.2 ± 47.2	191.7 ± 52.9	0.087
**Intraoperative blood loss (mL)**	111.7 ± 74.1	116.8 ± 82.1	0.125
**Number of lymph nodes removed**	44.2 ± 21.4	46.8 ± 18.1	0.286
**SMI (cm** ^**2**^ **/m** ^**2**^ **)**			
Preoperative	47.8 ± 7.6	38.9 ± 5.2	< 0.001
Postoperative	43.4 ± 7.2	36.6 ± 5.3	< 0.001
**Postoperative sarcopenia**			0.045
Yes	56 (37.1)	10 (21.3)	
No	95 (62.9)	37 (78.7)	
**Extent of resection (n**,** %)**			0.981
Distal gastrectomy	107 (70.9)	34 (72.3)	
Total gastrectomy	34 (22.5)	10 (21.3)	
Proximal gastrectomy	10 (6.6)	3 (6.4)	
**pT-stage (n**,** %)**			0.324
T1	46 (30.5)	16 (34.0)	
T2	51 (33.8)	12 (25.5)	
T3	36 (23.8)	9 (19.1)	
T4	18 (11.9)	10 (21.3)	
**pN-stage (n**,** %)**			0.868
N0	59 (39.1)	19 (40.4)	
N+	92 (60.9)	28 (59.6)	
**pTNM stage**^**#**^**(n**,** %)**			0.136
I	40 (26.5)	16 (34.0)	
II	48 (31.8)	19 (40.4)	
III	63 (41.7)	12 (25.5)	
**Tumor size (cm)**	5.4 ± 2.3	6.2 ± 2.3	0.413
**Histologic type (n**,** %)**			0.376
High-moderate differentiation	66 (43.7)	24 (51.1)	
Low-undifferentiated	85 (56.3)	23 (48.9)	
**Adjuvant chemotherapy (n**,** %)**			0.346
Yes	114 (75.5)	37 (78.7)	
No	37 (24.5)	10 (21.3)	

*Note* ASA, American Society of Anesthesiologists; p, pathologic; PNI, prognostic nutritional index; SMI, skeletal muscle index; BMI, Body mass index. #, The TNM stage was determined according to the 8th edition of the American Joint Committee on Cancer staging manual

### Changes in body composition during surgery

**Fig. 2:**
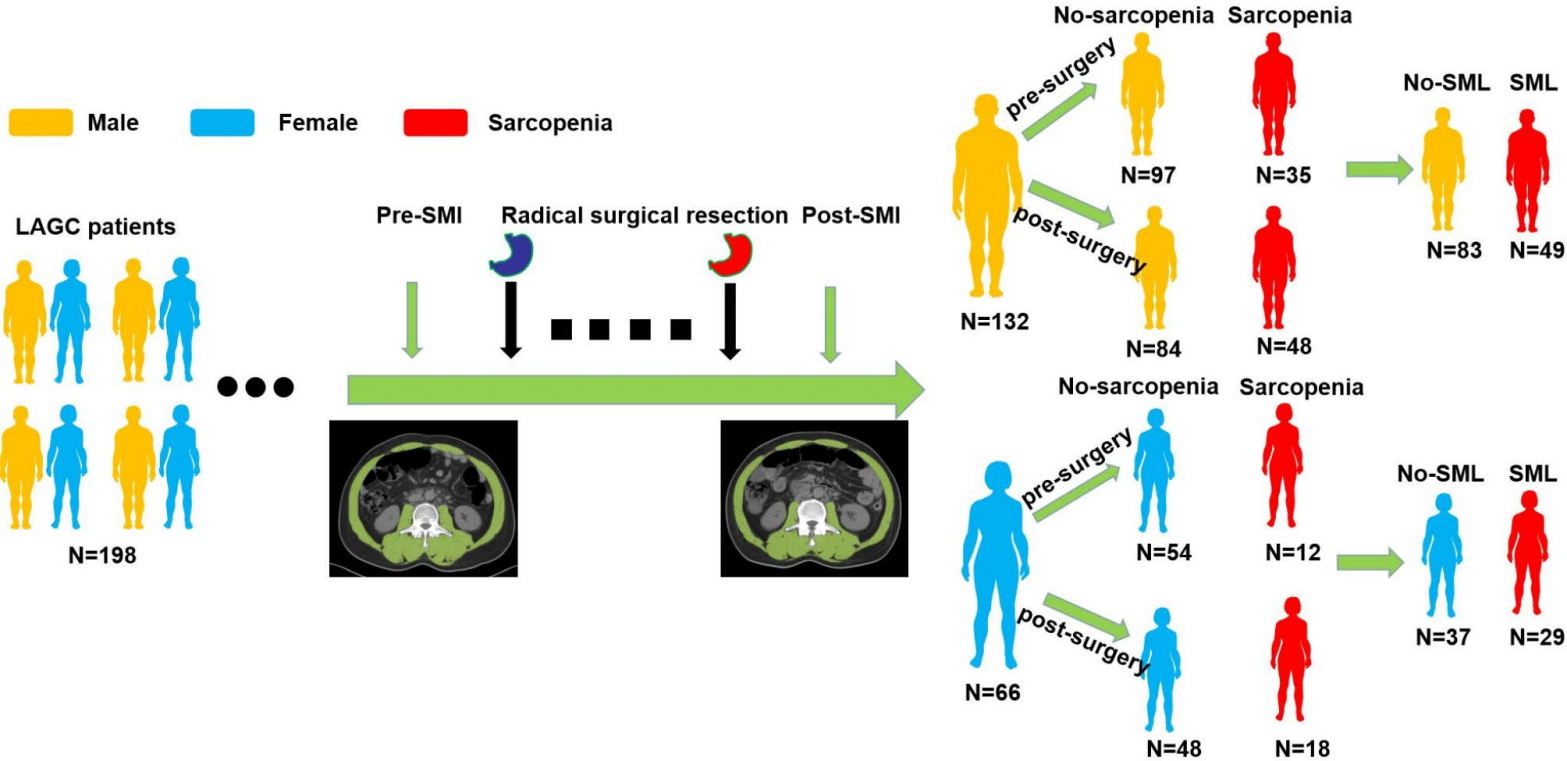
Perioperative Changes in Skeletal Muscle Status Among LAGC Patients. This diagram shows the classification and transitions of skeletal muscle status in 198 patients with LAGC before and after radical surgical resection. SMI was measured using CT imaging at the L3 vertebral level, and patients were initially stratified into No-sarcopenia and Sarcopenia groups based on AWGS 2019 guidelines (male: SMI < 40.8 cm^2^/m^2^; female: SMI < 34.9 cm^2^/m^2^). Postoperatively, significant muscle loss (SML) was defined as a decrease in SMI (%/30 days) exceeding 7.23% for men and 6.57% for women. Patients were further divided into No-SML and SML groups. Yellow figures represent male patients, and blue figures represent female patients

**Fig. 3:**
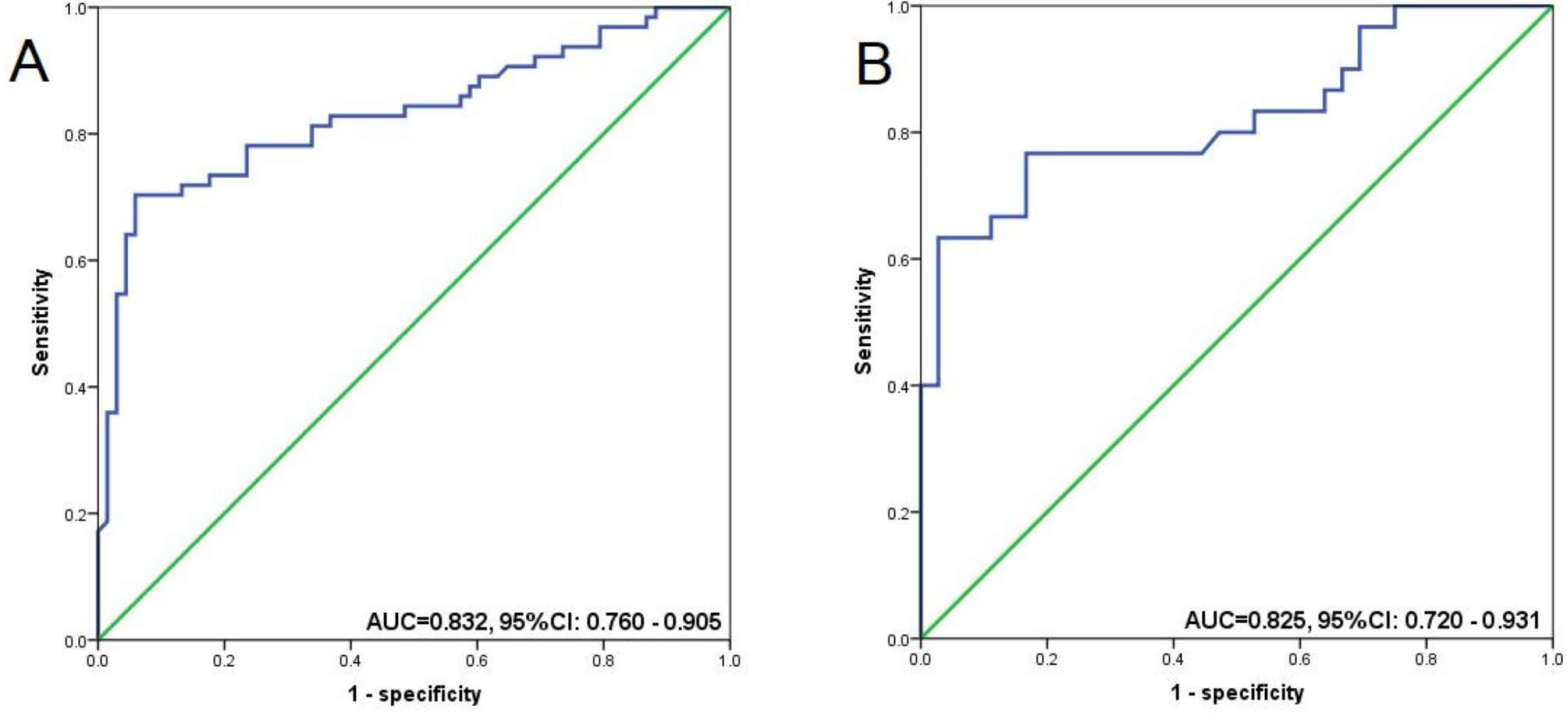
ROC curves for predicting SML and OS. The ROC curves evaluating the predictive performance of SMI changes for SML and OS in LAGC patients. The AUC values reflect the models’ accuracy, with optimal thresholds determined using Youden’s index, along with their sensitivity and specificity

Figure 2 illustrates changes in skeletal muscles at L3 under pre- and post-op conditions. At the second CT scan, the rate of sarcopenia was found to increase from 23.7% (47/198) at baseline to 33.3% (66/198) (*P* = 0.045). Notably, the mean loss in muscle quantity was not prominent in some cases as there was no gain or loss in muscle mass. The mean ΔSMI(%)/30 days of the cohort before and after radical surgical treatment was − 5.95 ± 3.50%. ROC curves were used wherever the optimal threshold value for ΔSMI(%)/30 days was not well-defined. For male subjects, the threshold value was 7.23% [area under the curve (AUC) = 0.832; 95%CI: 0.760–0.905], while in females, it was 6.57% (AUC = 0.825; 95%CI: 0.720–0.931) (Fig. 3). There were 78 individuals (39.39%) in the significant muscle loss (SML; male: ΔSMI(%)/30 days ≥ 7.23%; female: ΔSMI(%)/30 days ≥ 6.57%) group versus 120 (60.61%) subjects in the non-SML group.

### Impact of pre-op sarcopenia on post-op complications

According to the post-op follow-ups, 15 and 20 different complications were identified in the pre-op sarcopenic and non-sarcopenic groups, and the corresponding incidence rates were 31.91% and 13.25% (*P* = 0.003), respectively (Table 2). However, sarcopenic patients exhibited severely adverse post-op complications than their non-sarcopenic counterparts (CD score ≥ 3a; *P* = 0.014). While local complications were not significantly different between these groups. Eventually, post-op systemic complications, especially pulmonary complications, were significantly more common among pre-op sarcopenic individuals (*P* = 0.010).

**Table 2:** Comparisons of post-op complications [N(%)]

Post-op complications	Pre-surgery		*P*	Post-surgery		*P*	ΔSMI (%)/30 days		*P*
	Non-sarcopenia (*N* = 151)	Sarcopenia (*N* = 47)		Non-sarcopenia (*N* = 132)	Sarcopenia (*N* = 66)		Non-SML (*N* = 120)	SML (*N* = 78)	
**Any complications (n**,** %)**	20 (13.2)	15 (31.9)	0.003	19 (14.4)	16 (24.2)	0.087	13 (10.8)	22 (28.2)	0.002
**Clavien–Dindo classification (n**,** %)**									
I-II	15 (9.9)	9 (19.1)	0.091	11 (8.3)	8 (12.1)	0.394	10 (8.3)	8 (10.3)	0.646
III-IV	5 (3.3)	6 (12.8)	0.014	8 (6.1)	8 (12.1)	0.140	3 (2.5)	14 (17.9)	<0.001
**Any local complications (n**,** %)**	14 (9.3)	5 (10.6)	0.780	10 (7.6)	9 (13.6)	0.124	7 (5.8)	12 (15.4)	0.026
Wound problem	4 (2.6)	1 (2.1)	1.000	2 (1.5)	3 (4.5)	0.336	1 (0.8)	4 (5.1)	0.080
Anastomosis bleeding	2 (1.3)	2 (4.3)	0.239	1 (0.8)	3 (4.5)	0.109	2 (1.7)	2 (2.6)	0.647
Anastomosis leakage	1 (0.7)	1 (2.1)	0.419	0 (0.0)	2 (3.0)	1.000	0 (0.0)	2 (2.6)	1.000
Stump leakage	0 (0.0)	1 (2.1)	1.000	0 (0.0)	1 (1.5)	1.000	0 (0.0)	1 (1.3)	1.000
Post-op bleeding	2 (1.3)	1 (2.1)	0.559	1 (0.8)	2 (3.0)	0.258	0 (0.0)	3 (3.8)	1.000
Anastomosis obstruction	1 (0.7)	1 (2.1)	0.419	1 (0.8)	1 (1.5)	1.000	0 (0.0)	2 (2.6)	1.000
Pancreas fistula	1 (0.7)	0 (0.0)	1.000	0 (0.0)	1 (1.5)	1.000	0 (0.0)	1 (1.3)	1.000
Pancreatitis	1 (0.7)	1 (2.1)	0.419	0 (0.0)	2 (3.0)	1.000	0 (0.0)	2 (2.6)	1.000
Abdomen abscess	2 (1.3)	1 (2.1)	0.559	1 (0.8)	2 (3.0)	0.258	1 (0.8)	2 (2.6)	0.563
Intestinal obstruction	1 (0.7)	0 (0.0)	1.000	1 (0.8)	0 (0.0)	1.000	0 (0.0)	1 (1.3)	1.000
Adhesive ileus	1 (0.7)	2 (4.3)	0.141	2 (1.5)	1 (1.5)	1.000	1 (0.8)	2 (2.6)	0.563
Bowel stricture	0 (0.0)	2 (4.3)	0.055	1 (0.8)	1 (1.5)	1.000	0 (0.0)	2 (2.6)	1.000
Other local complications	3 (2.0)	4 (8.5)	0.056	4 (3.0)	3 (4.5)	0.688	2 (1.7)	5 (6.4)	0.115
**Any systemic complications (n**,** %)**	8 (5.3)	8 (17.0)	0.010	6 (4.5)	10 (15.2)	0.010	4 (3.3)	12 (15.4)	0.002
Pulmonary complication	5 (3.3)	7 (14.9)	0.008	4 (3.0)	8 (12.1)	0.022	3 (2.5)	9 (11.5)	0.009
Urinary complication	3 (2.0)	4 (8.5)	0.056	4 (3.0)	3 (4.5)	0.688	2 (1.7)	5 (6.4)	0.115
Other systemic complications	2 (1.3)	3 (6.4)	0.088	3 (2.3)	2 (3.0)	1.000	1 (0.8)	4 (5.1)	0.080

### Impact of underlying sarcopenia on the long-term prognosis in GC

**Fig. 4:**
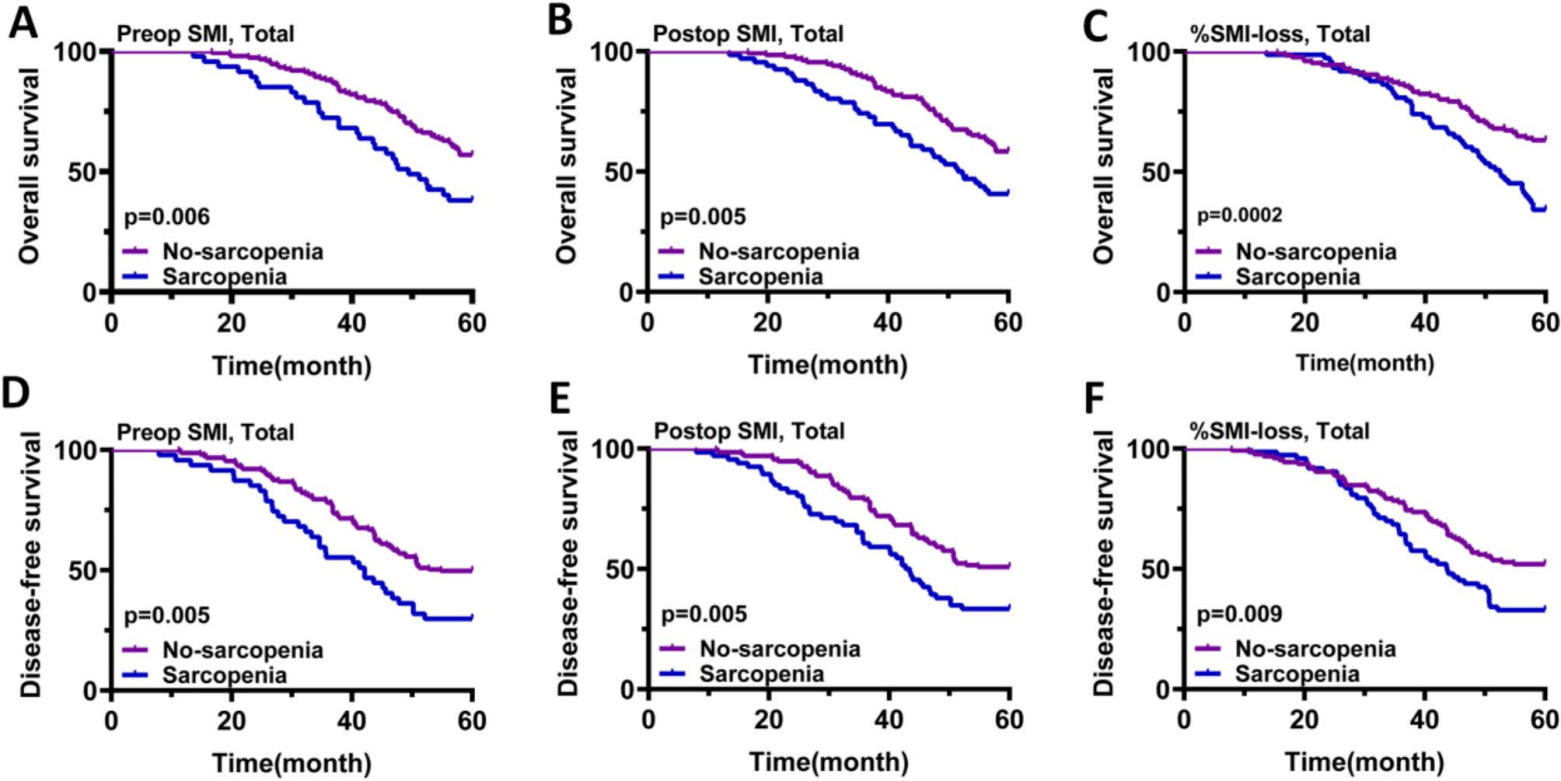
The Kaplan–Meier survival curves of OS and DFS based on the SMI in total cohort. (**A**) Effect of preoperative SMI on OS, (**B**) Effect of postoperative SMI on OS, (**C**) Effect of %SMI-loss on OS, (**D**) Effect of preoperative SMI on DFS, (**E**) Effect of postoperative SMI on DFS, (**F**) Effect of %SMI-loss on DFS

During 68.9 months (95%CI: 34.5–89.7) of median follow-ups, 94 (47.47%) patients died. Among these, 72 deaths (76.6%) were attributed to gastric cancer progression, 12 deaths (12.8%) were due to other malignancies, and 10 deaths (10.6%) were caused by non-cancer-related conditions. Additionally, during the follow-up period, we identified 78 cases of recurrence among LAGC patients. These included locoregional recurrence, which accounted for 23.1%, and distant metastasis, which constituted 37.2%. Within the distant metastasis group, liver metastasis accounted for 15.4%, lung metastasis for 10.3%, and bone metastasis for 11.5%. The most common pattern of recurrence was peritoneal recurrence, observed in 39.7% of cases. The 5-year OS rates of non-pre-op sarcopenic and pre-op sarcopenic cases were respectively 56.95% and 38.30% (*P* = 0.006; Fig. 4A).

Regarding post-op SMI, the non-post-op sarcopenia and post-op sarcopenia groups exhibited five-year OS rates of 63.20% and 34.25%, respectively (*P* = 0.005; Fig. 4B). The SML group candidates showed significantly worse OS in the KMS analysis (*P* = 0.002; Fig. 4C). When stratified by sex, both men and women exhibited a similar trend in the KPS curve in the entire cohort. Drastically worse OS rates were noticed in the pre- and post-op sarcopenia and SML groups (both *P* < 0.05; Supplementary Fig. 1). The correlative association between per-op SMI and DFS was similar to that of SMI and OS. The KMS curves for DFS revealed comparable results to that of OS curves (Fig. 4D-E, Supplementary Fig. 2).

In the multivariate Cox regression analysis, pre-op sarcopenia (OS: HR = 2.332, 95%CI: 1.219–8.231, *P* = 0.001; DFS: HR = 8.132, 95%CI: 1.523–36.523, *P* = 0.001), post-surgical sarcopenia (OS: HR = 3.189, 95%CI: 2.022–10.532, *P* = 0.011; DFS: HR = 3.023, 95%CI: 1.042–14.246, *P* = 0.003), and the onset of antitumor therapy-associated SML (OS: HR = 11.231, 95%CI: 2.532–31.221, *P* = 0.002; DFS: HR = 10.562, 95%CI: 2.312–40.022, *P* = 0.002) were independent risk factors for five-year DFS and OS in LAGC patients (Table 3, Supplementary Table 1). In addition, adjuvant chemotherapy was associated with a significant reduction in both the risk of death (HR = 0.532, 95%CI: 0.368–0.895, *P* = 0.015) for overall survival and the risk of recurrence (HR = 0.587, 95%CI: 0.342–0.982, *P* = 0.041) for disease-free survival.

In addition, we attempted to analyse the population included in this study into 4 groups based on changes in postoperative SMI: (1) those with normal SMI and no post-op decrease from pre-op (group A, *N* = 109); (2) those with normal pre-op SMI and a decrease (group B, *N* = 42); (3) those with low SMI before and possibly an improvement (group C, *N* = 11); (4) those with low SMI and no improvement or worsening after surgery (group D, *N* = 36). We further analysed the incidence of surgical complications in these four groups and found that there was no significant difference between groups B (11.65% vs. 4.76%, *P* = 0.175) and C (11.65% vs. 9.1%, *P* = 0.794) using group A as the reference standard, while the highest incidence was found in group D (11.65% vs. 47.6%, *P* < 0.001). We also analysed the 5-year OS as well as DFS between the four groups and found that patients in group D had the worst prognosis (5-year OS: 32.6%; 5-year DFS: 28.9%), whereas patients in group A (5-year OS: 64.2%; 5-year DFS: 61.6%) had the best, and those in groups B (5-year OS: 45.7%; 5-year DFS: 43.2%) and C (5-year OS: 49.8%; 5-year DFS: 46.7%) were comparable between the two.

**Table 3:** Cox multivariate analysis affecting 5-year OS and DFS in patients with LAGC

Variable	5-year OS multivariate analysis		5-year DFS multivariate analysis	
	HR (95%CI)	*P*	HR (95%CI)	*P*
**TNM stage**		0.001		0.001
II	Reference		Reference	
III	5.782 (2.892–12.434)		6.923 (2.092–14.223)	
**Sarcopenia**				
Non-Sarcopenic	Reference		Reference	
Pre-treatment Sarcopenia	2.332(1.219–8.231)	0.001	8.132(1.523–36.523)	0.001
Newly developed Sarcopenia	3.189(2.022–10.532)	0.011	3.023(1.042–14.246)	0.003
**ΔSMI(%)/30 days**		0.002		0.002
No-SML	Reference		Reference	
SML	11.231(2.532–31.221)		10.562(2.312–40.022)	
**Adjuvant chemotherapy**		0.015		0.002
No	Reference		Reference	
Yes	0.532 (0.368–0.895)		0.587 (0.342–0.982)	

*Abbreviations* ECOG, Eastern Cooperative Oncology Group; SML: Significant muscle loss; FCC: Free cancer cells

## Discussion

This study investigated the prognostic impact of perioperative sarcopenia and SML in patients with LAGC undergoing curative gastrectomy. Our findings revealed that both preoperative and postoperative sarcopenia, as well as SML, are strong independent predictors of poor OS and DFS. The prevalence of sarcopenia increased significantly after surgery, rising from 23.7% preoperatively to 33.3% postoperatively. Patients with SML not only exhibited worse survival outcomes but also experienced a higher incidence of severe postoperative complications (Clavien-Dindo grade ≥ 3a). Additionally, adjuvant chemotherapy was shown to significantly improve both OS and DFS, underscoring its critical role in the postoperative management of LAGC.

Patients with sarcopenia are less able to tolerate medical procedures, including surgery and chemotherapy. Recent studies have shown that people with measurable muscle loss may have shorter survival times [26–27]. However, there is no widely accepted way to measure skeletal muscle mass. Here, abdominal CT scans were applied to trace skeletal mass loss in these patients. Pre-op CT scans are frequently performed for clinical staging of GC, which gives CT imaging an advantage in assessing skeletal muscle to determine sarcopenia. A recent study has demonstrated an excellent connection between dual-energy X-ray absorptiometry (DXA) results and CT image analysis [28]. Hence, we propose that GC patients must undergo body composition analyses utilizing CT scans before surgery and adjuvant treatment, which also help identify sarcopenia condition.

Pre-op sarcopenia is recognized as an unfavorable prognostic indicator in GC, considering relatively longer hospital stays and in-hospital complications [29–30]. We demonstrated that pre-op sarcopenia was negatively correlated with serious post-op complications, especially pulmonary complications, which was inconsistent with previous research [31–32]. However, several studies have reported contradictory findings. Fang et al. have reported a cohort of 409 patients who underwent surgical resection for GC showed pre-op lower skeletal muscle mass, which was not significantly correlated with post-op complications [33]. Tegels et al. suggest that pre-op sarcopenia may not be associated with post-op morbidity and/or mortality [34]. The difference in results might be attributed to the different diagnostic procedures and criteria, as well as the patient’s demographic and clinical features, as a result of which the prevalence of sarcopenia in our study was different from these studies: 23.7% (47/198) versus 64.8% (265/409) and 57.7% (86/152), respectively. Therefore, determining the CT cut-off values of sarcopenia for different populations is highly desirable.

Recently, a growing number of studies suggest a negative impact of sarcopenia on OS, and the importance of skeletal muscle measurement in classifying cachexia and malnutrition [35]. Consistently, we noticed that pre-op sarcopenia could be an independent predictor for worse OS, however, the underlying mechanism remains enigmatic. Skeletal muscle loss could be induced by various factors, such as aging, hormonal imbalance, and inflammation [36]. The proliferation of breast cancer cells can be inhibited by cytokines secreted from muscle cells, and muscle mass loss may activate inflammatory pathways promoting tumor growth [37–38]. In this study, pre-op sarcopenia was correlated with a lower PNI in GC patients. Differences in survival outcomes between non-sarcopenic and pre-op sarcopenia individuals highlight the necessity of early diagnosis of sarcopenia to improve the 5-year survival rate.

Only a few studies have investigated the effect of post-op sarcopenia on survival [39–40]. GI surgery-related sarcopenia may directly affect survival by impairing immune activation and disrupting the metabolic system, resulting in abrupt muscle wasting [41]. A study by Naruji et al. reported that post-op skeletal muscle loss (> 5%) in GC patients at six months significantly shortened survival (*P* = 0.039) [39]. In the present study, we found different optimal thresholds using ROC curves for peri- and post-op changes in skeletal muscle mass based on the biological sex. With this criterion, 78 out of 198 LAGC patients experienced SML during the first month of surgery, suggesting that immediate post-surgical days may be crucial in intervening in skeletal muscle loss.

This study had several limitations. First, there were no objective criteria to characterize sarcopenia using other measures. Second, we couldn’t perform subgroup analyses of sarcopenia staging and secondary complications. Third, it was a single-center study retrospective study using a small study cohort. A prospective multicenter study in a larger cohort is required to further validate the implication of skeletal muscle loss in post-op outcomes in specific subsets of LAGC patients. Meanwhile, our study only included analyses of populations of East Asian descent. Future research should focus on replicating this study in different demographic settings to verify whether our results are universally applicable, thereby deepening our understanding of the role of sarcopenia on a global scale.

## Conclusions

Pre-operative sarcopenia is an independent predictor of short- and long-term clinical outcomes in LAGC patients receiving curative gastrectomy. Significant muscle loss (male: ΔSMI(%)/30 days ≥ 7.23%; female: ΔSMI(%)/30 days ≥ 6.57%) during gastrectomy and newly developed sarcopenia independently worsened OS and DFS. Future studies should explore the underlying cause of muscle degeneration and whether preventing muscle loss can improve clinical outcomes in GC patients.

## Electronic supplementary material

Below is the link to the electronic supplementary material.

Supplementary Material 1

## Data Availability

No datasets were generated or analysed during the current study.
