# Supplementary material for: Preoperative sarcopenia and postoperative accelerated muscle loss negatively impact survival after resection of locally advanced gastric cancer
Source: BMC Cancer. 2025 Feb 14;25:269. doi: 10.1186/s12885-025-13674-3 (PMC11829415; doi:10.1186/s12885-025-13674-3)
Supplement: Supplementary file 1 — Supplementary Material 1 [file 12885_2025_13674_MOESM1_ESM.docx]

**Supplementary Figures and Tables**

**
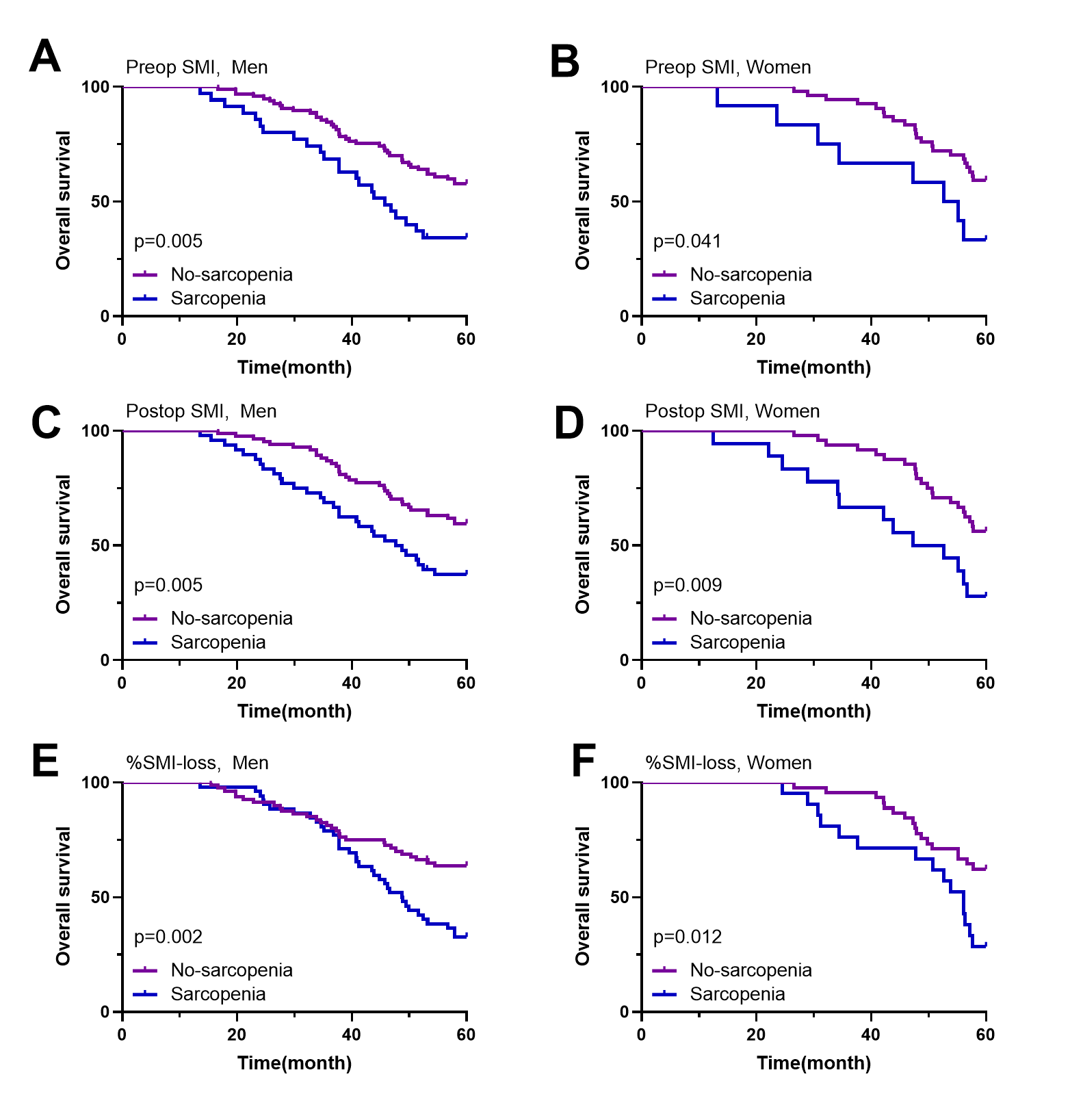
**

**Supplementary Figure 1. Kaplan-Meier survival curves of OS based on SMI in different genders. (A) Effect of preoperative SMI on OS in male patients, (B) Effect of preoperative SMI on OS in female patients, (C) Effect of postoperative SMI on OS in male patients, (D) Effect of postoperative SMI on OS in female patients, (E) Effect of %SMI-loss on OS in male patients, (F) Effect of %SMI-loss on OS in female patients.**

**
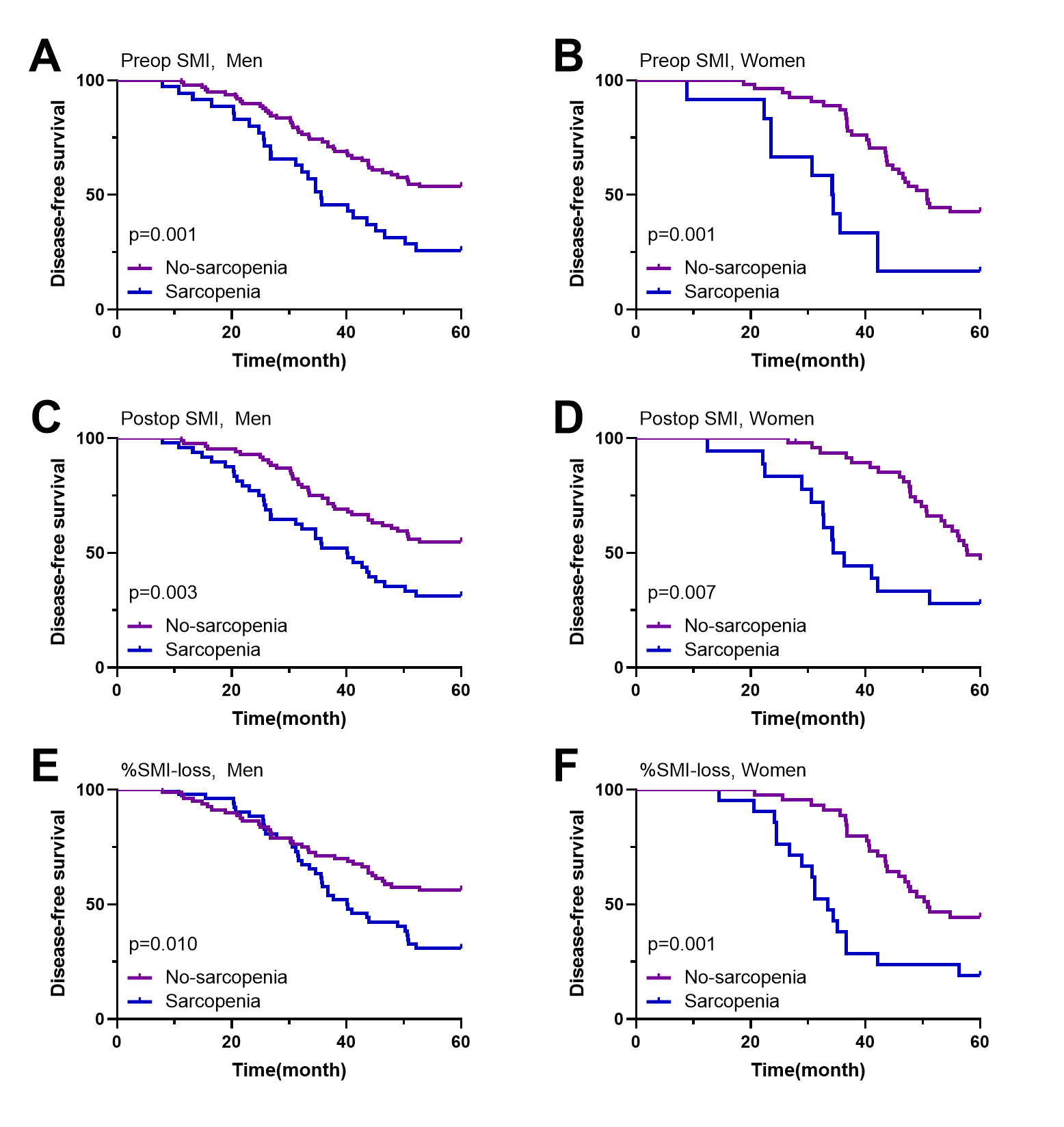
**

**Supplementary Figure 2. Kaplan-Meier survival curves of DFS based on SMI in different genders. (A) Effect of preoperative SMI on DFS in male patients, (B) Effect of preoperative SMI on DFS in female patients, (C) Effect of postoperative SMI on DFS in male patients, (D) Effect of postoperative SMI on DFS in female patients, (E) Effect of %SMI-loss on DFS in male patients, (F) Effect of %SMI-loss on DFS in female patients.**

**Supplementary Table 1. Cox univariate analysis affecting 5-year OS and DFS in patients with LAGC**

| **Variable** | **5-year OS univariate Analysis** | |  | **5-year DFS univariate Analysis** | |
| --- | --- | --- | --- | --- | --- |
|  | **HR (95%CI)** | **P** |  | **HR (95%CI)** | **P** |
| **Age (years)** |  | 0.131 |  |  | 0.659 |
| ≤50 | Reference |  |  | Reference |  |
| >50 | 0.451(0.122–1.611) |  |  | 0.821(0.324–2.561) |  |
| **ECOG** |  | 0.465 |  |  | 0.146 |
| 1 | Reference |  |  | Reference |  |
| 0 | 0.252(0.076–1.653) |  |  | 0.422(0.032–1.232) |  |
| **Gender** |  | 0.349 |  |  | 0.345 |
| Male | Reference |  |  | Reference |  |
| Female | 0.662(0.321–3.248) |  |  | 0.743(0.234–2.556) |  |
| **Lesion site** |  |  |  |  |  |
| Upper 1/3 | Reference |  |  | Reference |  |
| Middle 1/3 | 0.246(0.088–2.157) | 0.335 |  | 0.234(0.143–2.361) | 0.233 |
| Lower 1/3 | 0.672(0.091–3.322) | 0.461 |  | 0.571(0.023–2.395) | 0.436 |
| **Pre-BMI(Kg/m^2^)** |  | 0.464 |  |  | 0.523 |
| Underweight (<18.5) | Reference |  |  | Reference |  |
| Normal (≥18.5) | 0.641(0.329–4.434) |  |  | 0.651(0.012–2.230) |  |
| **Post-BMI(Kg/m^2^)** |  | 0.218 |  |  | 0.233 |
| Underweight (<18.5) | Reference |  |  | Reference |  |
| Normal (≥18.5) | 0.319(0.129–2.523) |  |  | 0.599(0.153–3.934) |  |
| **Borrmann type** |  | 0.792 |  |  | 0.410 |
| III–IV | Reference |  |  | Reference |  |
| I–II | 0.234(0.093–2.235) |  |  | 0.619(0.166–2.301) |  |
| **Histological** |  | 0.377 |  |  | 0.471 |
| Low | Reference |  |  | Reference |  |
| High-moderate | 0.609(0.123–3.342) |  |  | 0.643(0.148–2.797) |  |
| **pT staging** |  | 0.444 |  |  | 0.831 |
| T4 | Reference |  |  | Reference |  |
| T3 | 0.521(0.128–2.245) |  |  | 0.674(0.223–3.445) |  |
| **pN staging** |  | 0.431 |  |  | 0.746 |
| N+ | Reference |  |  | Reference |  |
| N0 | 0.632(0.146–2.321) |  |  | 0.832(0.457–2.642) |  |
| **Lesion size(cm)** |  | 0.234 |  |  | 0.934 |
| ≥5 | Reference |  |  | Reference |  |
| <5 | 0.647(0.144–2.425) |  |  | 0.866(0.234–2.870) |  |
| **Sarcopenia** |  |  |  |  |  |
| Non-Sarcopenic | Reference |  |  | Reference |  |
| Pre-treatment Sarcopenia | 3.233(1.280–50.520) | 0.001 |  | 8.539(3.354–34.980) | <0.0001 |
| Newly developed Sarcopenia | 6.740(2.321–39.032) | 0.015 |  | 7.939(1.464-11.632) | 0.011 |
| **ΔSMI(%)/30 days** |  | 0.000 |  |  | 0.001 |
| No-SML | Reference |  |  | Reference |  |
| SML | 17.031(3.028–48.840) |  |  | 11.734(4.028–51.03) |  |

**Abbreviations**: ECOG, Eastern Cooperative Oncology Group; SML: Significant muscle loss; FCC: Free cancer cells.
